# Child Abandonment, Developmental Harm, and Permanency Planning: A Systematic Review of Attachment, Trauma, and Termination of Parental Rights

**DOI:** 10.7759/cureus.113935

**Published:** 2026-08-04

**Authors:** Joy Ivaschenko

**Affiliations:** 1 Psychiatry, Christ Health Center Live Oaks, Birmingham, USA

**Keywords:** child abandonment, child rights, developmental harm, permanency, termination of parental rights

## Abstract

Child abandonment, delayed permanency, and prolonged reunification efforts continue to present complex challenges in child welfare decision-making. This systematic review synthesized current evidence regarding child abandonment, developmental trauma, attachment disruption, permanency planning, and termination of parental rights to evaluate whether existing evidence supports earlier permanency decisions when parental abandonment has been established.

A systematic review was conducted using MEDLINE, PubMed, Google Scholar, publications from the American Academy of Child and Adolescent Psychiatry, resources from the US Department of Health and Human Services, the Child Welfare Information Gateway, and additional US government reports and policy documents. Searches were conducted from March 2026 through June 5, 2026. English-language studies, governmental reports, statutes, policy documents, and peer-reviewed literature addressing abandonment, permanency, trauma, attachment disruption, developmental outcomes, and termination practices were included. Titles and abstracts were screened for relevance, followed by full-text review of eligible sources. Because of substantial heterogeneity in study designs, populations, legal frameworks, and outcome measures, statistical pooling was not appropriate. Findings were therefore synthesized using a structured qualitative narrative approach that grouped evidence into predefined thematic domains. Risk of bias for primary empirical studies was assessed using validated tools appropriate to study design (Newcastle-Ottawa Scale and Joanna Briggs Institute Critical Appraisal Checklists), while non-empirical sources were not formally appraised but were evaluated descriptively according to study design, source type, methodological rigor, and relevance. Data extraction included study design, population characteristics, legal or policy context, developmental outcomes, trauma-related outcomes, attachment-related findings, permanency outcomes, and implications for child welfare decision-making. A total of 333 records were identified, 12 duplicates were removed, 79 full-text articles and reports were assessed for eligibility, and 48 studies and reports were included in the final synthesis.

The reviewed literature consistently associated prolonged instability, caregiver deprivation, neglect, abuse, and abandonment with adverse developmental, psychological, and neurobiological outcomes. Earlier permanency was associated with more favorable developmental, emotional, and cognitive outcomes across the evidence reviewed. Overall, the findings support minimizing unnecessary delays in permanency decision-making when abandonment has been established. Although no scientifically validated time threshold for termination of parental rights was identified, the available evidence supports child welfare policies that prioritize timely permanency, child safety, stability, and long-term well-being.

## Introduction and background

Introduction

It is well established that federal and state laws require that "reasonable efforts" be made to reunify children with their parents, particularly their birth mothers, when safe and feasible [1,2]. However, the child welfare system, despite its intent to promote child safety and family preservation, is often understaffed and lacks uniform operational standards across jurisdictions [3]. This raises an important question: should reasonable efforts be required when a parent, or parents, has intentionally abandoned a child [4-6]?

Early permanency decisions are critical because childhood development is highly dependent on stable, responsive caregiving. When permanency is delayed, children may remain in prolonged states of uncertainty during periods of rapid brain development, increasing the risk of attachment disruption, toxic stress, and long-term psychological harm. Earlier placement into safe, permanent caregiving environments has consistently been associated with improved emotional, cognitive, and developmental outcomes.

Children who experience abandonment may struggle to develop or maintain a sense of safety and security in their attachment relationships. Accordingly, it is important to consider whether reunification under these circumstances promotes child safety or instead risks further disruption of attachment and emotional development, particularly when reunification is sought with the same parent who abandoned the child [7,8].

If clear and documented evidence exists of a parent's intent to abandon a child-whether through statements, actions, or documented communications, regardless of whether the parent is the mother or father-should there be a process that allows for termination of parental rights without parental consent [9-11]? Furthermore, if such termination is legally permissible, should the system require waiting six months when intent has already been established [12]? Should standards be strengthened to better protect children, and should permanency timelines be shortened to facilitate earlier placement in safe and stable homes? These questions highlight the broader policy tension between procedural safeguards and child-centered permanency needs [1,13].

These questions have important implications for child welfare practice because permanency timelines influence when courts, child protection agencies, and clinicians shift their focus from reunification toward establishing a permanent caregiving environment. Delayed decisions may unintentionally prolong children's exposure to instability, whereas timely permanency may reduce cumulative trauma and improve lifelong developmental trajectories.

Although substantial research exists regarding child maltreatment, trauma, attachment disruption, and permanency planning, no recent systematic review has specifically synthesized evidence relevant to abandonment, termination of parental rights, developmental trauma, and the potential consequences of delayed permanency decisions. Given the variability in abandonment standards across jurisdictions and the growing body of literature examining the developmental consequences of instability, a comprehensive review of the evidence is warranted.

Although closely related, abandonment, neglect, and caregiver instability represent distinct concepts that may coexist but are not synonymous. Abandonment refers to the intentional relinquishment of parental responsibilities through prolonged absence, failure to maintain contact or support, or demonstrated intent to permanently disengage from the parent-child relationship. Neglect refers to the ongoing failure to provide a child's physical, emotional, educational, or developmental needs, regardless of whether the caregiver remains physically present. Caregiver instability refers to repeated disruptions in caregiving relationships, placement changes, inconsistent caregiving, or prolonged uncertainty regarding who provides safe and reliable care. Each may independently contribute to developmental harm, but abandonment frequently encompasses elements of both neglect and caregiver instability.

Early childhood represents a particularly vulnerable developmental period where children are highly susceptible to the effects of toxic stress and adverse caregiving experiences [14]. Approximately 90% of brain development occurs before age five, when neural systems governing attachment, executive functioning, emotional regulation, learning, and stress responses are highly plastic and develop rapidly [15-17]. Exposure to abandonment, neglect, or prolonged caregiver instability during this period has been associated with altered brain architecture, insecure attachment, emotional dysregulation, psychopathology, and long-term impairments in cognitive and social functioning [7,14,15,18-20]. Stable caregiving is essential for healthy neurodevelopment.

Prolonged delays in permanency decisions are unlikely to be developmentally neutral and may allow preventable harm to accumulate during a critical period of brain development [14,15,18,21]. Evidence from statutory guidance governing involuntary termination of parental rights, together with trauma-informed care and resilience research, supports timely permanency planning when abandonment has been established [12,22-24]. Parents and caregivers play a fundamental role in promoting healthy child development, emotional security, and resilience through consistent, nurturing relationships [25]. The legal concept of a parent encompasses both the rights and the responsibilities inherent in the parent-child relationship, including the duty to provide care, protection, and support [26]. Accordingly, a child-centered standard requires clearly defined expectations of parental responsibility, particularly in abandonment cases in which caregiving responsibilities have been demonstrably discontinued [27,28]. When documented evidence of abandonment exists-such as written communication, recorded communication, or prolonged absence-the legal system should allow expedited termination procedures consistent with statutory definitions of abandonment [29].

Although no empirical evidence identifies a scientifically validated legal timeframe for permanency decisions following abandonment, these findings provide the developmental rationale for evaluating a proposed 30-60-day policy framework when abandonment has been clearly established. The lower limit is informed by evidence that posttraumatic stress disorder (PTSD) may be diagnosed after 30 days of persistent trauma symptoms and by existing statutory definitions recognizing abandonment after approximately 30 days in some jurisdictions [12,22,29]. Removal from an ongoing traumatic environment and placement into stable caregiving are associated with improved long-term developmental outcomes [8,23,24,30]. This recommendation is not presented as an evidence-based legal cutoff but rather as a child-centered policy framework derived from developmental neuroscience, attachment research, trauma literature, and existing statutory definitions, several of which already recognize abandonment after approximately 30 days [7,14,22,29-31].

If one parent remains and actively provides safe and consistent care, and no concerns exist regarding safety or developmental risk, that parent should be granted full parental rights, while termination should proceed for the parent who has abandoned the child [25,28]. This approach aligns with established legal definitions of parental responsibility and abandonment across multiple jurisdictions [4-6].

Taken together, these findings underscore the importance of aligning permanency decision-making with developmental science by minimizing unnecessary delays following verified abandonment and promoting timely resolution of caregiving uncertainty [30,31]. Timely permanency supports children's fundamental need for safety, stability, and consistent caregiving rather than extending efforts to preserve parental rights that are no longer being exercised.

Prioritizing permanency aligns with the intent of the Adoption and Safe Families Act (ASFA), which emphasizes placing children in safe, stable, and permanent environments rather than allowing prolonged uncertainty that adversely affects development [1,32]. This framework is further supported by trauma-informed care models, which emphasize preventing chronic stress exposure during critical developmental periods [22,33].

The purpose of this systematic review is to synthesize the available evidence regarding child abandonment, attachment disruption, developmental trauma, permanency planning, and termination of parental rights and to evaluate whether current developmental, psychological, and policy evidence supports earlier permanency decisions when abandonment has been clearly established. This review further seeks to inform legislative and clinical decision-making by examining the developmental consequences of prolonged instability and evaluating evidence supporting defined permanency timelines, including expedited permanency for verified abandonment and time-limited opportunities for parental rehabilitation when active efforts toward reunification are being demonstrated.

Materials and methods

This systematic review examined the literature related to child abandonment, reunification, permanency planning, developmental outcomes associated with unstable caregiving environments, trauma, attachment disruption, legal statutes, child welfare policy, developmental psychology, public health guidance, and termination of parental rights.

Search Strategy

Literature searches were conducted using MEDLINE, PubMed, Google Scholar, publications from the American Academy of Child and Adolescent Psychiatry, resources from the US Department of Health and Human Services, publications from the Child Welfare Information Gateway, and additional US government reports, statutes, and policy documents. Searches were conducted from March 2026 through June 5, 2026.

Search terms included combinations of the following keywords: "termination of parental rights," "childhood abandonment," "abandonment and termination of parental rights," "statistics regarding parental abandonment," "reasonable efforts," "reunification," "permanency planning," "child trauma," "adverse childhood experiences," "attachment disruption," "caregiver deprivation," "child neglect," "child abuse," "posttraumatic stress disorder," and "child welfare policy." Searches were conducted using combinations of Boolean operators (AND/OR) and database-specific syntax as appropriate. Search strategies were adapted to the indexing structure of each database while maintaining the same conceptual search domains.

Searches were conducted in MEDLINE, PubMed, Google Scholar, and selected governmental and professional organization websites between March 2026 and June 2026. Medical Subject Headings (MeSH), when available, and keyword combinations were adapted to each database using Boolean operators (AND/OR). Search concepts included child abandonment, parental abandonment, attachment, attachment disruption, trauma, toxic stress, adverse childhood experiences (ACEs), permanency, termination of parental rights, child welfare, foster care, neurodevelopment, resilience, and developmental outcomes. Because search functionality varies across databases and governmental websites, search syntax was modified as appropriate while maintaining consistent conceptual domains. To facilitate interpretation of the synthesized evidence, a conceptual evidence-to-policy translation model was developed to integrate the principal legal, developmental, and clinical findings identified during the review.

**Figure 1:**
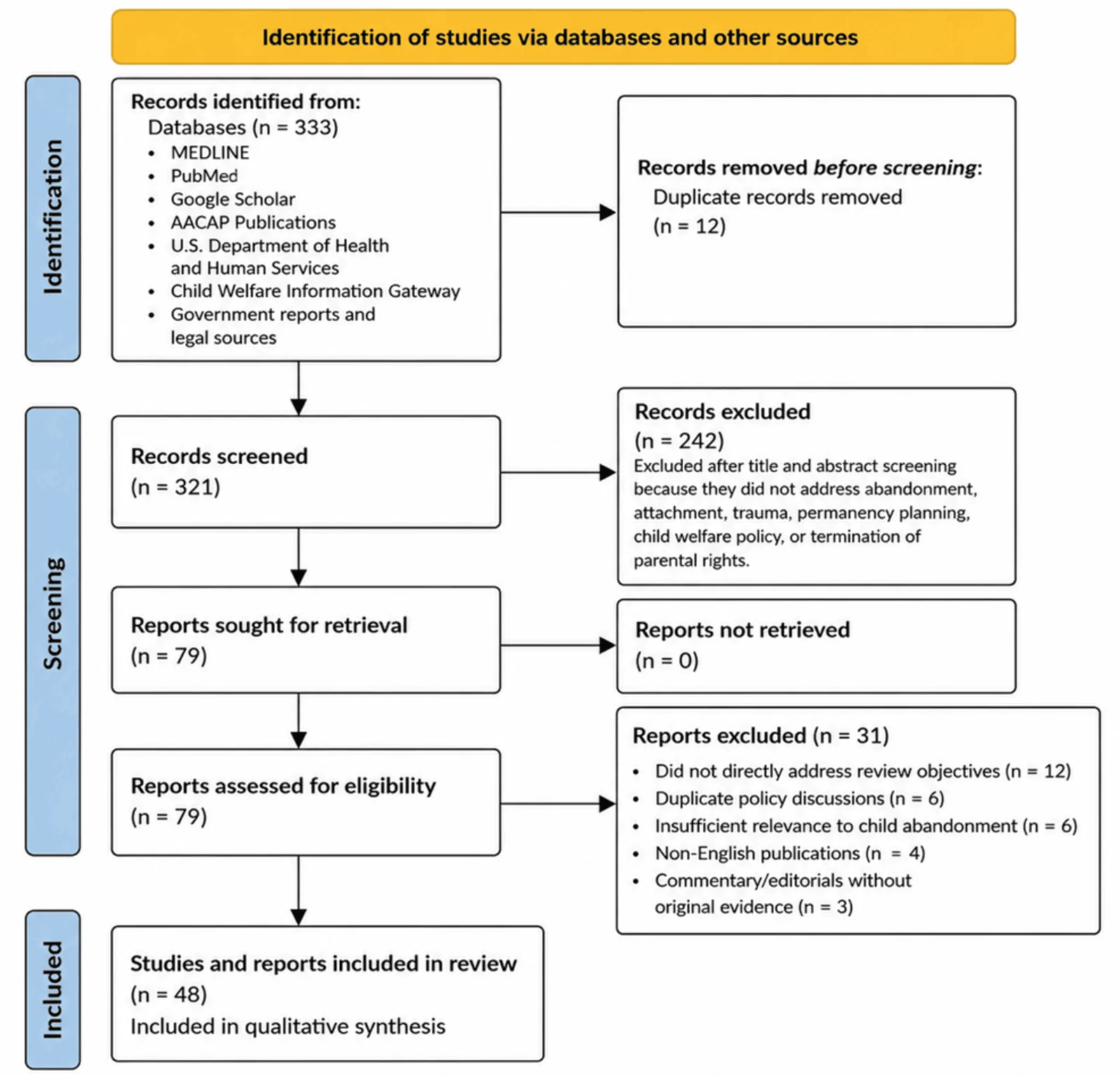
PRISMA 2020 Flow Diagram Flow diagram illustrating identification, screening, eligibility assessment, and inclusion of studies in accordance with the Preferred Reporting Items for Systematic Reviews and Meta-Analyses (PRISMA) 2020 statement. Literature searches identified 333 records from electronic databases, governmental publications, and legal sources. After removal of 12 duplicates, 321 records underwent title and abstract screening. Seventy-nine full-text reports were assessed for eligibility, of which 48 studies and reports met the predefined inclusion criteria and were included in the qualitative synthesis. AACAP: American Academy of Child and Adolescent Psychiatry

The study identification, screening, eligibility assessment, and inclusion process are summarized in Figure 1.

Data Synthesis and Conceptual Integration

The conceptual evidence-to-policy translation model synthesized legal, developmental, neurobiological, and clinical evidence identified during the review. The model integrates statutory definitions of abandonment, developmental science related to attachment and toxic stress, and empirical trauma literature to illustrate the pathway from documented abandonment to developmental outcomes and permanency policy implications.

Table 1 represents this evidence-to-policy translation model developed from the findings of this systematic review. Rather than depicting a single causal pathway, the model synthesizes current legal, developmental, neurobiological, and trauma evidence into a framework intended to inform child welfare policy and clinical decision-making. The complete literature search strategy, databases searched, and search terms used for this review are summarized in Table 1.

**Table 1:** Evidence-to-Policy Translation Model: Child Abandonment, Developmental Impact, and Permanency Decision Pathways Evidence-to-policy translation model illustrating the pathway from statutory abandonment definitions through developmental and neurobiological mechanisms to child outcomes and permanency policy implications. The model integrates legal frameworks, attachment theory, developmental neuroscience, toxic stress research, and trauma epidemiology to demonstrate how delayed permanency decisions may contribute to adverse developmental outcomes and how timely permanency may support improved child well-being. The conceptual framework was developed by the author based on evidence synthesized from the literature reviewed, including attachment theory [7], toxic stress and early brain development [14], attachment disruption in early deprivation [8], and developmental trauma [31]. ASFA: Adoption and Safe Families Act; PTSD: posttraumatic stress disorder

A. Legal trigger layer	Abandonment defined in statutes and policy frameworks (e.g., ASFA, state abandonment statutes, and termination of parental rights guidelines).
↓	
B. Behavioral event	Documented parental abandonment, including explicit refusal, prolonged absence, or verified communication indicating intent to abandon.
↓	
C. Developmental mechanism layer	Attachment disruption, toxic stress activation, and altered neurodevelopmental processes during early childhood developmentally sensitive periods.
↓	
D. Clinical & long-term outcomes	Increased risk of PTSD, anxiety, depression, behavioral dysregulation, substance use, and suicidality associated with prolonged instability.
↓	
E. Moderating factor: time exposure	Duration of instability as a dose-dependent risk factor, with heightened vulnerability in early childhood (particularly before age 5).
↓	
F. Policy response node	Permanency decision framework evaluating expedited termination timelines when abandonment is clearly established (e.g., 30-60 days).
↓	
G. Outcome: child-centered permanency	Stable attachment formation, reduced trauma exposure, improved developmental trajectory, and long-term psychological stability.

Eligibility Criteria

Studies were eligible for inclusion if they (1) were published in English; (2) were peer-reviewed empirical studies, systematic reviews, meta-analyses, governmental reports, professional guidance documents, or relevant legal authorities; (3) addressed child abandonment, attachment, neglect, developmental trauma, permanency planning, termination of parental rights, ACEs, or related developmental outcomes; and (4) provided information relevant to the objectives of this review. Studies were excluded if they were non-English publications, editorials, commentaries without substantive evidence, conference abstracts lacking sufficient methodological detail, duplicate publications, or articles not directly relevant to the review objectives.

Study Selection

Two-stage screening was conducted by reviewing titles and abstracts followed by full-text review of potentially eligible articles. Titles and abstracts were screened for relevance by the author. Potentially eligible studies underwent full-text review using the predefined eligibility criteria. Reasons for exclusion at the full-text stage included failure to address the review question, lack of relevant developmental or child welfare outcomes, duplicate reporting, or insufficient methodological information. The study selection process is summarized in the Preferred Reporting Items for Systematic Reviews and Meta-Analyses (PRISMA) flow diagram. Studies meeting the predefined inclusion criteria were included in the qualitative synthesis. Because this review included diverse evidence types, formal methodological quality assessment was performed for empirical studies and evidence syntheses (systematic reviews and meta-analyses) using validated tools appropriate to study design. Narrative reviews, legal statutes, policy documents, clinical guidelines, government reports, and theoretical papers were included for contextual synthesis but were not eligible for formal risk-of-bias assessment because validated appraisal instruments are not available for these evidence types.

Data Extraction

For each included source, data were extracted using a standardized approach that included author, year of publication, study design, population or data source, primary exposure or intervention, outcome measures, principal findings, and relevance to child abandonment, attachment, trauma, permanency, or child development. Information from legal authorities, policy documents, and professional guidance was extracted according to the legal or policy issue addressed. Extracted data were reviewed for consistency before narrative synthesis.

Risk-of-Bias Assessment

Methodological quality was assessed only for primary empirical studies using validated appraisal instruments appropriate to study design. Prospective and retrospective cohort studies were evaluated using the Newcastle-Ottawa Scale (NOS) [34], analytical cross-sectional and observational studies were assessed using the Joanna Briggs Institute (JBI) Critical Appraisal Checklist for Analytical Cross-Sectional Studies [35], and systematic reviews and meta-analyses were evaluated using the AMSTAR-2 instrument [36].

Narrative reviews, theoretical papers, professional guidelines, government reports, legal statutes, and policy documents were not formally assessed because validated risk-of-bias instruments are not designed for these evidence types. Because validated risk-of-bias instruments are not available for narrative reviews, theoretical articles, legal statutes, professional guidelines, or policy documents, these evidence sources were not formally scored but were evaluated descriptively according to methodological rigor, authority, and relevance to the review objectives. Results of the quality assessments are presented in Table 2.

**Table 2:** Risk-of-Bias Assessment of Empirical Studies

Study	Design	Tool	Score	Overall risk
Zeanah et al. [8]	Prospective cohort	Newcastle-Ottawa Scale	9/9	Low
Teicher and Samson [18]	Systematic review	AMSTAR-2	High confidence	Low
Widom et al. [20]	Prospective cohort	Newcastle-Ottawa Scale	8/9	Low
Russotti et al. [21]	Longitudinal cohort	Newcastle-Ottawa Scale	8/9	Low
Schofield et al. [24]	Meta-analysis	AMSTAR-2	Moderate-high confidence	Low
Narayan et al. [28]	Longitudinal cohort	Newcastle-Ottawa Scale	7/9	Low
Felitti et al. [37]	Retrospective cohort	Newcastle-Ottawa Scale	8/9	Low
Diamond-Welch and Kosloski [38]	Cross-sectional	Joanna Briggs Institute (JBI)	7/8	Low
Ben-David [39]	Observational	Joanna Briggs Institute (JBI)	7/8	Low
Davies et al. [40]	Longitudinal cohort	Newcastle-Ottawa Scale	8/9	Low
Ogelman [41]	Cross-sectional	Joanna Briggs Institute (JBI)	5/8	Moderate
Runcan [42]	Observational	Joanna Briggs Institute (JBI)	3/8	High
Iosim et al. [43]	Cross-sectional	Joanna Briggs Institute (JBI)	6/8	Moderate
Marici et al. [44]	Cross-sectional	Joanna Briggs Institute (JBI)	5/8	Moderate

Risk-of-bias assessments were independently completed by the author using published scoring criteria for each validated instrument. NOS scores of 7-9 stars were interpreted as low risk of bias, 5-6 as moderate risk, and lower than 5 as high risk. JBI assessments were interpreted according to the proportion of appraisal criteria satisfied, while AMSTAR-2 confidence ratings followed published guidance. Individual appraisal judgments for each empirical study are summarized in Table 2, together with the appraisal instrument applied according to study design. The methodological quality ratings for each empirical study are summarized in Table 2.

Data Synthesis

Due to substantial heterogeneity in study designs, populations, legal jurisdictions, outcome measures, and evidence types, statistical pooling and meta-analysis were not considered appropriate. Instead, a structured qualitative narrative synthesis was performed. Extracted data were reviewed iteratively and grouped into predefined thematic domains based on the review objectives, including legal standards, abandonment definitions, attachment disruption, developmental outcomes, trauma-related outcomes, permanency planning, and child welfare policy (Appendix). Findings were then compared across empirical studies, systematic reviews, governmental reports, and legal sources to identify areas of consistency, divergence, and evidence gaps. Greater emphasis was placed on findings supported across multiple study designs and higher-quality evidence as determined by the risk-of-bias assessment.

Theoretical frameworks reviewed included the "reasonable efforts" standard and child-centered permanency approaches. A quantitative meta-analysis was not attempted because substantial clinical, methodological, and conceptual heterogeneity precluded meaningful statistical pooling of effect estimates. Because quantitative synthesis was not performed, subgroup analyses, sensitivity analyses, and meta-regression were not applicable. Statistical software was not required because no quantitative synthesis or meta-analysis was undertaken.

## Review

Results

Epidemiology and Legal Standards

There is currently no standardized timeframe across states for determining abandonment. One legal definition includes an unexcused absence of 30 days, from which further policy recommendations will later be made [2,12].

In Utah, abandonment is defined as intentional relinquishment of custodial responsibilities with failure to reestablish them after 30 days, which may result in termination of parental rights [29]. Other definitions extend this period to six months without contact or presence in the child's life. Frequently cited standards require four to six months without communication [2,12]. All of this confirms the lack of consistent standards and thus a needed recommendation for standardized timeframes.

Risk-of-Bias Assessment

Methodological quality was assessed for all empirical studies using validated instruments appropriate to study design. Cohort and longitudinal studies were evaluated using the NOS, a validated tool developed by Wells et al.; analytical cross-sectional and observational studies were assessed using the JBI Critical Appraisal Checklist for Analytical Cross-Sectional Studies; and systematic reviews and meta-analyses were evaluated using AMSTAR-2 [34-36].

Six cohort or longitudinal studies achieved NOS scores ranging from 7 to 9 of 9 possible stars, indicating overall low risk of bias. Two evidence syntheses evaluated with AMSTAR-2 demonstrated high methodological confidence with no critical weaknesses identified. Cross-sectional and observational studies evaluated using the JBI checklist demonstrated greater variability, with one study rated as high risk of bias because of methodological limitations, three studies demonstrating moderate risk, and two studies demonstrating low risk. Common limitations included observational study designs, limited adjustment for confounding variables, convenience sampling, and restricted external validity.

Overall, the empirical evidence was judged to be of low-to-moderate risk of bias. Although causal inference remains limited because most included studies were observational, the consistency of findings across multiple study designs, populations, and independent investigations increases confidence in the overall conclusions.

Research demonstrates that children may experience significant developmental and psychological harm after as little as three days to two weeks of abandonment. This raises an important question: should timelines for termination be shortened to better protect children and promote stability? If a child remains in an environment characterized by ongoing maltreatment throughout multiple developmental stages, their ability to achieve psychopathologically relevant developmental milestones is more likely to be significantly impaired [21].

Although time alone is not typically sufficient to establish abandonment, despite its disastrous consequences, additional criteria include lack of contact, including persistent failure to communicate with or engage in the child's daily life/care, absence of financial support, lack of a parent-child relationship, expressed intent to relinquish parental responsibility, and absence without justification [6]. Whether each of these indicators should carry equal legal weight and support termination within 30 to 60 days (shortest timeframe selected because of first definition for abandonment and developmental consequences) when abandonment has been clearly established remains an important policy question.

The lack of consistency across jurisdictions contributes to delays and prolonged instability for children. A standardized timeframe of 30 to 60 days, particularly when parental intent is clearly demonstrated, may improve consistency in decision-making and facilitate earlier movement toward permanency. This standardization across jurisdictions would also create more generalized stability for children. Further research is needed to determine the optimal timeframe; however, existing evidence strongly supports minimizing unnecessary delays once abandonment has been established.

Legal and Child Welfare Context

In certain circumstances, reasonable efforts are not required when a parent has abandoned or abused a child [2]. Abandonment is not just limited to a single act but may include ongoing patterns of emotional and physical withdrawal, withholding affection and love, lack of engagement with the child when needed, and failure to meet a child's daily physical and emotional needs [12,19,22].

One child welfare law enforcement manual defines abandonment as a parent's demonstrated intent to terminate the parent-child relationship through restricting communication, failing to provide care, or failing to provide appropriate supervision. Nominal or superficial attempts at contact are generally not considered sufficient to maintain the parent-child relationship [12]. Similarly, Romanian law defines abandonment as the failure to meet a child's basic needs [42].

Neglect, abuse, and rejection-defined as the failure to provide appropriate care, attention, or emotional support, the infliction of harm to a child's well-being, or refusal of child or child-centered emotional/psychological support-are closely associated with abandonment [43,45,46]. According to the American Psychological Association, neglect is the failure to provide a child with necessary care, attention, emotional support, love, and affection, often resulting from indifference, inadequate caregiving, or caregiver impairment due to substance use or other causes, thereby interfering with normal development [42]. Child abuse encompasses acts or omissions that result in physical and/or psychological harm [46].

Legal inconsistencies, particularly in cases involving medical abandonment, further highlight the need for clearer standards. While some newborn cases result in rapid adoption proceedings, other situations lack clearly defined timelines, contributing to prolonged instability.

One statute states: "The failure of a parent to provide reasonable support and to maintain regular contact with the child, including providing normal supervision... includes a judicial finding that a parent has made only minimal efforts to support and communicate with the child. Failure to maintain a normal parental relationship with the child without just cause for a period of six months constitutes prima facie evidence of abandonment" [9].

Abandonment of a child within a medical facility is often defined by parental actions rather than a specific statutory timeframe. When a parent fails to retrieve a child from a medical facility after discharge, legal authorities may become involved; however, this action alone does not necessarily result in permanent legal consequences [5,11,12]. The absence of clearly defined timelines in these situations may contribute to prolonged instability for the child and raises questions regarding whether time-based standards should also be established for medical abandonment. In some jurisdictions, newborns may be placed for adoption within two to four days when a parent fails to retrieve the infant following hospital discharge and legal intervention has occurred. If consent for termination of parental rights can be obtained from the known parent, including the father when legally identified, termination may occur within this timeframe to facilitate timely adoption [12,13,32].

Developmental and Psychological Impact

Evidence consistently demonstrates that abandonment, caregiver deprivation, instability, and neglect are associated with adverse developmental and psychological outcomes. Abandonment generally involves the intentional relinquishment of a child without the intent to return and frequently includes the severing of emotional attachments [44]. Such abandonment constitutes voluntary relinquishment of parental responsibilities and, consequently, parental rights. Evidence indicates that emotional abandonment during early childhood-particularly by the primary caregiver, most notably the maternal caregiver-may produce measurable developmental effects within days to weeks, with long-term consequences extending throughout childhood and adulthood [7,30]. Attachment theory research identified abandonment as a significant disruption to the development of secure caregiver-child relationships. Children exposed to caregiver absence, emotional unavailability, or prolonged instability demonstrated increased rates of attachment disturbances, emotional dysregulation, anxiety, and relational difficulties throughout childhood and adulthood [7,30,47].

Insufficient caregiver communication, responsiveness, and emotional support during early childhood are associated with long-term emotional, cognitive, and relational difficulties. According to the National Center for PTSD, acute stress disorder (ASD) symptoms may emerge within three days following a traumatic event, whereas PTSD may be diagnosed when symptoms persist beyond 30 days. Although research regarding ASD continues to evolve, early identification, prompt intervention, and removal of the precipitating source of trauma are considered important in reducing the risk of persistent psychological injury. Research supports that trauma-related symptoms may therefore develop within days rather than months or years [48].

These findings raise important questions regarding current abandonment standards that often require children to remain in uncertain caregiving situations for four to six months, or longer, before legal intervention may occur. If trauma symptoms may emerge within days and become clinically significant within weeks, should permanency decisions continue to be delayed for months when parental intent to abandon has already been clearly established [48]?

There is also a strong correlation between early childhood trauma and poorer psychological outcomes following subsequent traumatic experiences [48]. Early childhood represents a uniquely vulnerable developmental period. Neural systems responsible for attachment, emotional regulation, executive functioning, and stress responses undergo rapid development during this time, making young children particularly susceptible to the effects of abandonment, neglect, and prolonged instability [14,15].

When a primary caregiver, particularly a maternal caregiver during early childhood, fails to provide consistent love, nurturing, and responsiveness, children are at increased risk for long-term developmental, emotional, and relational difficulties. Children whose primary caregiver is emotionally unavailable or inconsistently responsive may develop emotional dysregulation, difficulty trusting caregivers, and impaired attachment security. Children also develop emotional issues and an inability to rely on caregivers appropriately when the maternal caregiver lacks responsiveness to their needs or presence. Some evidence further suggests that these attachment disruptions may increase vulnerability to subsequent exploitation or revictimization later in life. Children who have a parent, particularly the mother, who dismisses them and their needs experience high levels of trauma from it that causes lasting mental, relational, and physical issues. Children whose emotional and developmental needs are consistently dismissed experience increased psychological distress and long-term mental, relational, and physical health consequences [40,41].

Abandonment with parental re-entry following prolonged absence has been associated with anxiety, impaired attachment formation, emotional instability, and other trauma-related symptoms that may persist throughout the lifespan [2,4,16,30,47]. Children from birth through six years of age are particularly vulnerable to developmental disruption. Maltreatment during this period has been associated with failure to achieve developmental milestones, altered brain development, and long-term impairment in the ability to regulate emotional and social stressors in as much as 90% of children [18]. The neurological consequences of prolonged instability are well documented. Chronic exposure to stress elevates cortisol levels and has been associated with measurable structural and functional changes in the amygdala, hippocampus, and prefrontal cortex-brain regions responsible for emotional regulation, memory, impulse control, and executive functioning. Children exposed to prolonged neglect or instability are at increased risk for emotional dysregulation, anxiety disorders, depression, behavioral disorders, and trauma-related symptoms that may persist into adulthood [14,15].

The long-term consequences become even more concerning when viewed through the lens of ACEs. The landmark ACE Study found that individuals exposed to four or more ACEs had approximately a 460% increased risk of depression and up to a 1,220% increased risk of suicide attempts compared with individuals reporting no ACE exposure. Parental substance use disorder frequently contributes to ACE exposure and is therefore an important consideration in many termination of parental rights proceedings [39]. Additionally, exposure to multiple childhood adversities is strongly associated with substance misuse, behavioral disorders, vulnerability to sexual exploitation, and numerous adverse health outcomes throughout the lifespan [37,38,49].

The methodological quality ratings for each empirical study are presented in Table 2. The types of evidence included in the review are summarized in Table 3.

**Table 3:** Types of Included Evidence

Evidence type (representative references)	Number of sources
Primary empirical research studies [8,20,21,29,35,37,40,41,46-48]	16
Systematic reviews/meta-analyses/review articles [16-19,24,25,28,43]	10
Government reports and agency publications [2,3,12,15,22,33,39]	9
Professional organization reports/guidance [23,26,34,49]	4
Legal statutes and legislation [1,4-6,9-11,30]	8
Legal/reference sources [27]	1
Total	48

Non-empirical sources that were included for contextual synthesis but were not eligible for formal risk-of-bias assessment are summarized in Table 4.

**Table 4:** Non-empirical Evidence Included for Contextual Synthesis These sources provided theoretical, legal, policy, and contextual information that informed the qualitative synthesis but were not eligible for formal methodological quality or risk-of-bias assessment because validated appraisal instruments are not available for these evidence types. This approach is consistent with recommendations for systematic reviews that include mixed evidence sources.

Source	Evidence type	Formal bias assessment
Bowlby [7]	Theoretical framework	Not applicable
Nelson et al. [16]	Narrative review	Not applicable
Marshall and Kenney [17]	Narrative review	Not applicable
Cicchetti and Toth [19]	Narrative review	Not applicable
Reading et al. [27]	Narrative review	Not applicable
Masten and Monn [23]	Narrative review	Not applicable
World Health Organization (WHO) [33]	Guideline	Not applicable
American Psychological Association (APA) [25,50]	Professional guidance	Not applicable
Child Welfare Information Gateway [2,3,12]	Government report	Not applicable
State statutes [1,4-6,9-11,29]	Legal authority	Not applicable

Widom et al. reported that children with documented abuse or neglect histories had approximately 2.5-fold greater odds of developing major depressive disorder than non-maltreated controls (reported effect estimate and 95% confidence interval) and were nearly twice as likely to develop anxiety disorders compared with non-maltreated children. They were also significantly more likely to develop trauma-related disorders later in life [20]. These findings are highly suggestive that delays in permanency decisions are not neutral and are likely to contribute to additional developmental, emotional, and psychological harms. The longer children remain without a stable, committed caregiver, the greater their risk for long-term emotional, relational, and psychological difficulties, supporting the need for clear, timely permanency decisions following confirmed abandonment.

Evidence from the Bucharest Early Intervention Project further supports the importance of timely permanency. Children exposed to prolonged caregiver deprivation demonstrated severe attachment disturbances, whereas those placed into stable caregiving environments at younger ages experienced substantially improved emotional, cognitive, and developmental outcomes. Children placed before two years of age demonstrated the greatest developmental gains, suggesting that reducing the duration of instability may substantially improve long-term outcomes [8].

Research additionally suggests that chronic neglect may be among the most damaging forms of child maltreatment. Neglect has been associated with significant deficits in language development, executive functioning, emotional regulation, and cognitive performance and, in some studies, predicts poorer long-term outcomes than isolated episodes of physical abuse [18]. This finding is particularly relevant in abandonment cases because abandonment frequently involves prolonged emotional neglect even prior to the caregiver’s departure, absence of responsive caregiving, and failure to meet a child's developmental needs.

Consistent, stable, engaged, and responsive caregivers-whether biological, adoptive, or otherwise-provide the stable environment necessary for healthy child development. Children placed in stable, supportive homes more quickly demonstrate developmental recovery and continued developmental gains [17]. Consistent caregiving promotes emotional well-being, academic achievement, resilience, adaptive coping, empathy, healthy peer/social relationships, and optimal neurodevelopment. Research demonstrates that caregiver responsiveness and consistency-not biological relatedness alone-are the primary determinants of positive developmental outcomes [7,50]. These findings reinforce the importance of achieving permanent, stable caregiving arrangements as soon as it becomes evident that a biological parent is unwilling or unable to fulfill that role.

Conversely, abandonment during childhood has been associated with impaired self-esteem, difficulty trusting others, chronic insecurity within relationships, anxiety, depressive symptoms, avoidance of close relationships, fear of abandonment, and increased vulnerability to unhealthy interpersonal relationships. Abandonment during any stage of childhood has been associated with adverse mental health outcomes in adulthood, with younger children demonstrating the greatest vulnerability [19,23].

These findings further support minimizing unnecessary delays in permanency decisions, as prolonged caregiver instability increases the likelihood of enduring emotional and psychological harm. Providing children with caregivers who remain consistently present, responsive, and committed offers the greatest opportunity for healthy developmental outcomes [50].

Key quantitative findings identified during the review and their implications for child welfare decision-making are summarized in Table 5.

**Table 5:** Research Findings Supporting Consideration of Earlier Permanency Decisions When Abandonment Intent Is Established Current evidence strongly supports the harms associated with prolonged instability, caregiver deprivation, neglect, and delayed permanency. However, the literature does not establish a universally accepted scientific threshold for termination of parental rights. The proposed 30-60-day timeframe should therefore be viewed as a policy recommendation informed by developmental, attachment, trauma, and child welfare research rather than a specific empirically validated cutoff. PTSD: posttraumatic stress disorder

Research finding	Statistical evidence	Relevance to child welfare decision-making
Acute stress response following trauma [23]	Symptoms of acute stress disorder may develop within 3 days of a traumatic event.	Demonstrates that psychological harm can begin long before many current abandonment timelines expire.
Posttraumatic stress development [23,26]	PTSD may be diagnosed when trauma symptoms persist beyond 30 days.	Suggests that clinically significant trauma symptoms may emerge during the period when many systems continue reunification efforts.
Early brain development [14,15]	Approximately 90% of brain development occurs before 5 years of age.	Highlights the importance of minimizing instability during critical periods of neurological development.
Effects of toxic stress [14-18]	Chronic stress alters neural pathways involved in emotional regulation, attachment, executive functioning, and stress responses.	Indicates that prolonged instability may contribute to lasting developmental and mental health consequences.
Adverse childhood experiences (ACEs) and depression [20,21,39,41]	Exposure to 4 or more ACEs is associated with an approximately 460% increased risk of depression.	Demonstrates the cumulative impact of repeated childhood adversity.
ACEs and suicidality [20,21,39,41]	Exposure to 4 or more ACEs is associated with up to a 1,220% increased risk of suicide attempts.	Illustrates the potential lifelong consequences of unresolved childhood trauma.
Abuse and neglect outcomes-depression [20,21]	Children with documented abuse or neglect histories are approximately 2.5 times more likely to develop depression.	Suggests that continued exposure to instability may increase psychiatric morbidity.
Abuse and neglect outcomes-anxiety disorders [20,21]	Children with documented abuse or neglect histories are approximately 2 times more likely to develop anxiety disorders.	Demonstrates significant long-term mental health risks associated with maltreatment.
Institutional deprivation and attachment [8,31,43]	Children exposed to prolonged caregiver deprivation demonstrated severe attachment disturbances.	Supports the importance of timely access to stable, responsive caregiving relationships.
Early placement into stable caregiving [24,25,29,34]	Children placed into stable caregiving environments before age 2 showed substantially improved cognitive and emotional outcomes.	Suggests that reducing time spent in unstable environments may improve developmental outcomes.
Chronic neglect [19-21,37]	Neglect is associated with significant deficits in language development, executive functioning, emotional regulation, and cognitive performance.	Demonstrates that inaction and prolonged uncertainty may themselves contribute to developmental harm.
Placement stability [8,24,25,29,34,40]	Stable and permanent caregiving relationships are associated with improved emotional, behavioral, cognitive, and social outcomes.	Supports policies aimed at reducing unnecessary delays in achieving permanency.

*Trauma-Related Outcomes* 

Research consistently demonstrated that trauma-related symptoms may emerge rapidly following adverse experiences. These findings indicate that significant psychological harm may occur long before many existing abandonment timelines expire [48]. Studies further demonstrated strong associations between childhood adversity and long-term mental health outcomes, including depression, anxiety disorders, substance misuse, suicidality, and trauma-related disorders.

Discussion

Interpretation of Findings

This systematic review identified consistent evidence demonstrating that abandonment, caregiver deprivation, prolonged instability, neglect, and delayed permanency are associated with adverse developmental, psychological, and neurobiological outcomes. Although most empirical studies demonstrated moderate to high methodological quality, the evidence base was largely observational and therefore remains susceptible to residual confounding and limitations inherent to non-randomized study designs.

Because of marked heterogeneity in study design, study populations, outcome measures, legal definitions of abandonment, and inclusion of both empirical and non-empirical evidence sources, quantitative pooling of results was neither methodologically appropriate nor consistent with PRISMA guidance for mixed-evidence systematic reviews. Consequently, findings were synthesized narratively using predefined thematic domains rather than pooled statistical estimates.

Despite inherent limitations associated with observational research, the consistency of findings across longitudinal cohorts, systematic reviews, cross-sectional studies, developmental neuroscience, and child welfare research strengthens confidence that supports the conclusion that the observed associations between abandonment, prolonged instability, and adverse developmental outcomes are unlikely to be explained solely by systematic bias.

Trauma-related findings were particularly noteworthy. Evidence demonstrated that symptoms of ASD may emerge within days following a traumatic event, while clinically significant trauma symptoms may develop within weeks.

These findings raise important questions regarding abandonment standards that often require children to remain in uncertain circumstances for months or even years before permanency decisions may occur. While the literature does not establish a definitive abandonment timeline, the evidence consistently demonstrates that developmental and psychological harm may occur long before many existing legal timelines expire.

Implications for Child Welfare Practice

One of the principal policy frameworks identified throughout the literature is the concept of "reasonable efforts," which requires child welfare agencies to make legally mandated efforts to prevent the removal of a child or facilitate reunification whenever it can be accomplished safely [2]. Although these requirements appropriately emphasize family preservation, the evidence synthesized in this review raises important questions regarding whether current implementation consistently balances parental rights with children's developmental, emotional, and physical well-being.

The evidence reviewed suggests that prolonged instability, repeated disruptions in caregiving, and delayed permanency decisions contribute to adverse developmental and psychological outcomes that persist into adulthood [7,14,18,20]. While reasonable efforts are intended to provide a stable and supportive environment for healthy child development [2], the literature raises important questions regarding whether continued reunification efforts consistently achieve this objective when one or both parents have demonstrated an intent to permanently disengage from the child's life through abandonment.

When considering reasonable efforts, it is necessary to evaluate whether the system is appropriately balancing parental rights with a child's right and need for safety, consistency, stability, and emotional, mental, and physical well-being. If a parent has demonstrated an unwillingness to fulfill parental responsibilities through abandonment, continued reunification efforts may not serve the child's best interests. In such circumstances, reasonable efforts may appropriately be shifted toward achieving timely permanency while continuing to prioritize child safety and developmental well-being. Establishing clear and standardized timelines for permanency determinations may help ensure that the primary focus remains on providing children with stable and secure caregiving environments rather than prolonging uncertainty in an effort to preserve parental rights that are no longer being exercised. From developmental and clinical perspectives, minimizing prolonged instability is consistent with evidence demonstrating that secure, stable caregiving relationships are among the strongest protective factors for healthy childhood development [14,23].

Implications for Child Welfare Policy

The evidence reviewed highlights the ongoing challenge of balancing parental rights with children's developmental needs. The findings further suggest that periods of prolonged instability should not be viewed as neutral waiting periods. Rather, evidence indicates that developmental, emotional, and psychological harm may continue to accumulate when children remain without stable and permanent caregiving arrangements. Studies examining institutional deprivation, foster care instability, neglect, and attachment disruption consistently demonstrate improved outcomes when children achieve permanency earlier and experience permanent stable caregiving environments [7,8,14,18]. Across the reviewed literature, stable caregiving relationships were consistently associated with improved emotional, behavioral, cognitive, and social outcomes, whereas instability, caregiver absence, and uncertainty were associated with poorer developmental outcomes. Accordingly, when there is clear evidence of abandonment, timely assessment of parental intent and caregiving capacity should guide decision-making, with the focus shifting toward permanency rather than prolonged reunification efforts. Within this context, preventing prolonged instability and repeated disruptions in caregiving may reduce trauma exposure and adverse developmental outcomes [14,31].

Reintroducing a parent after abandonment, particularly without clear and sustained evidence of change, may retraumatize the child and create further instability. When reunification cannot be demonstrated to be safe or developmentally beneficial, the evidence supports consideration of termination of parental rights and placement in a stable, permanent caregiving environment, whether through adoption or with the remaining parent who has consistently provided appropriate care.

These findings support child-centered permanency approaches that prioritize safety, stability, and developmental well-being. When parental abandonment has been clearly established, timely evaluation of permanency options may better align with developmental and trauma research than extended periods of uncertainty. This is particularly relevant when another parent or caregiver is already providing a safe, stable, and appropriate home environment.

The findings also highlight substantial variability in abandonment definitions and timelines across jurisdictions. Some states recognize abandonment after relatively brief periods of demonstrated absence or relinquishment, whereas others require substantially longer periods before legal action may occur. Greater consistency in abandonment standards may improve decision-making and reduce unnecessary delays in permanency planning.

In contrast, permanent placement-whether with a stable biological caregiver or adoptive caregiver(s)-supports healing, secure attachment, and long-term development. Termination of the abandoning parent's parental rights, when supported by statutory criteria and the child's best interests, may allow children to establish a consistent and secure caregiving environment.

The current lack of standardized timelines and consistent enforcement may create systemic gaps that prioritize parental rights over child stability. A more child-centered approach recognizes that stability, consistency, and permanency are fundamental components of healthy childhood development. Legal and policy frameworks should reflect the growing body of developmental and trauma research supporting these principles.

The evidence synthesized in this review also supports consideration of greater consistency in statutory definitions of abandonment and permanency timelines across jurisdictions. Clearly and consistently implemented decision-making timeframes-such as evaluation of documented abandonment within 30 to 60 days when evidence demonstrates an intent to abandon-may reduce variation in practice and help ensure that children are not left in prolonged periods of instability while legal proceedings continue.

At its core, child welfare policy should prioritize children's developmental needs. The evidence indicates that children require stable, permanent, and emotionally secure caregiving environments to achieve optimal developmental outcomes, regardless of whether those caregivers are biological or adoptive.

While current systems emphasize reunification, the evidence reviewed suggests that prolonged instability itself produces lasting developmental harm, suggesting reunification practices should be reconsidered. Children deserve timely decisions that prioritize their need for safety, stability, and permanence. Establishing clear standards and evidence-informed timelines based on trauma symptom development and a child's developmental well-being for abandonment determinations and permanency planning represents one potential approach to better protecting the long-term well-being of vulnerable children.

If the other parent continues to provide safe, stable, and developmentally appropriate care, and no concerns exist regarding the child's welfare, consideration may be given, consistent with applicable law and the child's best interests, to award sole legal parental rights to that parent while terminating the parental rights of the parent who abandoned the child. As previously discussed, stable caregiving has been associated with secure attachment formation, emotional regulation, resilience, cognitive development, academic achievement, healthy interpersonal relationships, and positive long-term mental health outcomes [25].

Additional research is needed to further define evidence-based standards of care and optimal permanency timelines following parental abandonment. Continued investigation may help inform future legislation, clinical practice, and child welfare policy aimed at protecting children's long-term developmental, psychological, and emotional well-being [2,7,12,14,31].

Limitations

This review has several limitations. First, relatively little research directly examines abandonment-specific timelines for termination of parental rights. Most available evidence is derived from the broader literature on trauma, attachment disruption, neglect, caregiver deprivation, ACEs, and permanency planning. As a result, conclusions regarding abandonment timelines should be interpreted within the broader developmental and child welfare context.

Second, risk-of-bias assessments were completed by a single reviewer using validated appraisal instruments rather than by multiple independent reviewers, which may introduce subjective judgment despite the use of standardized assessment criteria. Third, the included studies varied substantially in methodology, study populations, legal definitions, and outcome measures. The included studies differed substantially in design, populations, outcome measures, legal frameworks, and evidence type, preventing meaningful statistical pooling or meta-analysis.

Fourth, the review incorporated governmental reports, policy documents, legal statutes, and child welfare guidance in addition to peer-reviewed research. Although these sources provide important context regarding child welfare practice and legal standards, they do not always undergo the same methodological review processes as empirical studies.

Fifth, definitions of abandonment differed considerably across jurisdictions and studies. This lack of standardization complicates interpretation of the findings and limits the ability to identify universally applicable thresholds for abandonment determinations.

Sixth, the review was restricted to English-language publications and selected databases (MEDLINE, PubMed, Google Scholar, and governmental and professional organization resources). Relevant studies indexed exclusively in databases such as PsycINFO, Embase, Scopus, or Web of Science may therefore not have been identified. Finally, although validated appraisal tools were applied according to study design, much of the evidence originated from observational studies, policy reports, and legal literature, limiting causal inference and contributing to overall heterogeneity.

Future Research

Additional research is needed to better understand the relationship between abandonment timelines, permanency decisions, and child outcomes. Future studies should examine whether specific durations of abandonment are associated with differences in developmental, psychological, behavioral, neurobiological, and social outcomes. Prospective and longitudinal studies comparing child outcomes across varying permanency timelines would strengthen the evidence base and help inform evidence-based policy.

Further investigation is also needed to determine how abandonment should be defined across jurisdictions and how parental intent, duration of absence, lack of communication, and failure to provide care should be weighted in legal decision-making. The development of standardized definitions, outcome measures, and reporting frameworks would strengthen future research, improve comparability across studies, and facilitate more robust evidence synthesis. Future studies should also evaluate whether proposed permanency evaluation timeframes, including the evidence-informed 30-60-day framework described in this review, improve developmental and child welfare outcomes compared with existing practices.

## Conclusions

The evidence reviewed demonstrates that parental abandonment, caregiver deprivation, prolonged instability, neglect, and delayed permanency are associated with adverse developmental, psychological, and neurobiological outcomes. In contrast, stable, responsive, and permanent caregiving relationships represent among the strongest protective factors supporting healthy attachment, emotional regulation, resilience, and long-term child well-being. The literature reviewed further synthesized in this review indicates that prolonged uncertainty and instability should not be viewed as neutral periods of waiting, as developmental harm may continue to accumulate when children remain without consistent and secure caregiving environments. Although current research does not establish a scientifically validated timeline or universal threshold for termination of parental rights, the combined evidence from developmental science, attachment research, trauma literature, and child welfare studies supports minimizing unnecessary delays in permanency decision-making when abandonment has been clearly established. Child welfare systems should continue to balance parental rights with the fundamental need to protect children while recognizing that stability, consistency, and permanence are critical components of healthy development.

By integrating developmental science, trauma research, child welfare policy, and legal considerations, this review provides an evidence-informed framework for future legislative, judicial, and clinical decision-making regarding parental abandonment and permanency planning. Additional prospective and longitudinal research is needed to establish evidence-based permanency timelines following abandonment. Until such evidence is available, the proposed 30-60-day framework should be viewed as an evidence-informed policy recommendation rather than a validated legal standard. An evidence map summarizing the principal thematic domains and supporting literature is shown. Aligning child welfare practices with developmental science may improve long-term health, safety, and well-being for vulnerable children.

## Appendices

An evidence map summarizing the principal thematic domains and supporting literature is provided in Table 6.

**Table 6:** Evidence Map by Thematic Domain This evidence map summarizes the principal conceptual domains identified in the qualitative synthesis and the corresponding references that informed each thematic area. Several references contributed to more than one domain because they addressed multiple aspects of child development, trauma, attachment, child welfare policy, and permanency planning. ASFA: Adoption and Safe Families Act

Thematic domain	Conceptual focus	Supporting references
Legal and policy framework	Reasonable efforts, abandonment definitions, termination standards, ASFA intent	[1-6,9-13]
Attachment theory	Caregiver-child bonding, attachment disruption, abandonment effects on trust and emotional security	[7,8,26,28,31,38,43]
Neurodevelopment	Brain architecture, toxic stress, stress-response systems, sensitive periods, neurodevelopmental consequences	[14-18,32]
Trauma and adverse childhood experiences (ACEs)	Psychological harm, toxic stress, long-term physical and mental health outcomes, cumulative adversity	[18-23,39,41]
Attachment disruption and caregiving instability	Separation, neglect, institutional care, caregiver inconsistency, developmental effects	[8,16,29,35,48]
Permanency and resilience	Stable caregiving, protective factors, resilience, permanency outcomes, recovery	[23-25,34]
Social and intergenerational effects	Lifespan consequences, parental abandonment, intergenerational transmission of adversity, social functioning	[29,40,42,45,46]
Policy synthesis	Permanency timelines, child-centered decision-making, ASFA alignment, proposed 30-60-day permanency framework	[1-3,12,14,34]
